# Developing attributes for discrete choice experiments in health: a systematic literature review and case study of alcohol misuse interventions

**DOI:** 10.3109/14659891.2015.1118563

**Published:** 2016-03-10

**Authors:** Timea Mariann Helter, Christian Ernst Heinrich Boehler

**Affiliations:** ^a^ Health Economics Research Group, Brunel University, Middlesex, UK; ^b^ European Commission, Joint Research Centre, Institute for Prospective Technological Studies, Seville, Spain

**Keywords:** Alcohol misuse, discrete choice experiments, systematic review

## Abstract

Discrete choice experiments (DCEs) become increasingly popular to value outcomes for health economic studies and gradually gain acceptance as an input into policy decisions. Developing attributes is a key aspect for the design of DCEs, as their results may misguide decision-makers if they are based on an inappropriate set of attributes. However, the area lacks guidance, and current health-related DCE studies vary considerably in their methods of attribute development, with the consequent danger of providing an unreliable input for policy decisions. The aim of this article is to inform the progress toward a more systematic approach to attribute development for DCE studies in health. A systematic review of the published health-related DCE literature was conducted to lay the foundations for a generic framework which was tested in a case study of alcohol misuse interventions. Four stages of a general attribute development process emerged: (i) raw data collection; (ii) data reduction; (iii) removing inappropriate attributes; and (iv) wording. The case study compared and contrasted a qualitative and mixed-methods approach for the development of attributes for DCEs in the area of alcohol misuse interventions. This article provides a reference point for the design of future DCE experiments in health.

## Background

Discrete choice experiments (DCEs) have become increasingly popular to value outcomes for economic studies of health-related interventions. They consist of hypothetical scenarios with competing alternatives, characterized by a limited set of varying attributes (Gerard et al., 2008). Hence, the validity of DCEs depends on how complex information about health policies or interventions is transformed into a limited number of relevant attributes (Mangham et al., 2009). Coast et al. (2012) highlighted that “*if important choices are excluded because the research team did not think they were important, or if respondents do not fully understand the meaning of the attributes they are faced with, then, intuitively and self-evidently, the resulting DCE is likely to be biased or even useless for policy formation”* (p. 731). Conversely, if one uses more attributes than appropriate in a given context, the DCE might become too complex for respondents and might fail to elicit preferences (Lancsar & Louviere, 2008).

Previous studies highlight the lack of rigor of conducting and reporting attribute development (Grewal et al., 2006; Coast & Horrocks, 2007; Louviere & Lancsar, 2009). Coast et al. (2012) emphasized that biased or useless results of a DCE are “*inevitably, more likely when the process for developing attributes lacks a meticulous and rigorous approach”* (p. 732). Our article aims to inform the progress toward a more systematic approach to attribute development for health-related DCEs. To reduce complexity, we treat attribute development distinct from level selection for attributes, though we acknowledge that both tasks are intrinsically tied to each other. First, we systematically reviewed the literature to find out why and how researchers developed or suggested to develop attributes in health-related DCEs. We then aimed to identify common principles in attribute development that could lay the foundations for a generic framework. Finally, we tested the findings of our literature review in a case study of alcohol misuse interventions. A public health area was chosen due to the identified need to value the non-health outcomes of public health interventions (NICE, 2009). Alcohol misuse interventions were selected to demonstrate the development of attributes as they are likely to have both positive and negative impacts beyond health.

## Literature search

### Methods

Our systematic review of the health-related DCEs identified why and how researchers developed or suggested to develop attributes, the methods used (or suggested), and how such decisions were justified. English and German language studies published between 1990 and 2011 were included. Search terms comprised commonly used DCE terminology (e.g., conjoint analysis, choice experiment) and different combinations of the expression “developing attributes.” Databases searched included CINAHL, SCOPUS, PsychInfo, Cochrane Database of Systematic Reviews, Health Technology Assessment Database, NHS EED, and Ethos for relevant PhD theses. In addition, grey literature was searched in Google.com.

Studies were selected for inclusion in a two-stage process: First, papers were excluded after sifting titles and abstracts. Second, relevant studies were included after full-text review if they provided information on how attributes were developed, or suggestions related to this matter. Empirical DCEs and methods papers were fully reviewed, whilst discussion sections of literature reviews were searched for relevant information and further studies.

Each attribute development method used or suggested in the reviewed literature was considered and we used the software package NVivo for subsequent data analysis. The analysis process may best be described as an iterative approach with constant digesting and reviewing of the data, with the purpose of identifying emerging themes from the raw data. This iterative process continued until a basic structure emerged that could lay the foundations for a generic framework that describes the development of attributes in health-related DCEs and which could then be tested in a case study.

### Results

After sifting titles and abstracts of 3147 papers and reviewing 111 papers in full text, we found 86 health-related DCEs that used or suggested methods for developing attributes.

Attribute development was mostly considered a multistep process; however, the steps described varied depending on which part of this process researchers placed their particular emphasis on. For instance, Günther et al. (2010) used a “*multistep funnel-shaped process”* (p. 216), starting with a brainstorming task and followed by a stepwise reduction of attributes; Coast et al. (2012) recommend that attribute development should be divided into “*first, conceptual development and second, refinement of language to convey the intended meaning”* (p. 735). Coast and Horrocks (2007) followed an iterative approach where “*data collection and analysis proceed concurrently”* (p. 26) and reported some, but not all, stages of their attribute development process. In addition, a range of methods for attribute development was identified, and though there was some overlap in methods, they were applied at different stages of the attribute development process. For instance, focus groups were used to collect raw data about the most important aspects of the decision problem by Ratcliffe and Longworth (2002), to reduce the potential list of attributes by Youngkong et al. (2010) and to confirm the wording of selected attributes by Bell et al. (2010)

Hence, an “overriding principle” of attribute development did not immediately emerge as the literature reviewed used or suggested different methods in different sequences. Therefore, we further analyzed the information extracted, whilst focusing on emerging themes that support the understanding of the different stages of attribute development in health-related DCEs. Consequently, four distinct stages emerged to which different methods and suggestions relate: (i) raw data collection; (ii) data reduction; (iii) removing inappropriate attributes; and (iv) wording of attributes. This sequence follows the thought that, after initial data collection, there is a need to structure and reduce data which, as evidence suggests, is often vast and scattered. The resulting long list of potential attributes should then be screened for appropriateness based on the required characteristics, whose wording is then finalized during the final step of attribute development. Table 1 displays the proposed multistage process, and it also assigns methods reported or suggested to define attributes to each step within this process, including a brief description of methods and an exemplary study for further reference.

**Table 1.** Proposed multistage process of attribute development and potential methods.

Stage	Potential methods or approaches to reduce data		Examples of methods used or suggested		Brief definitions
Stage 1: RAW DATA COLLECTION	Qualitative methods	Focus groups	Coast et al. (2012)		Group of people are asked about their perceptions, opinions, beliefs, and attitudes toward the area being investigated
		Patient interviews			Conversation with a patient where the interviewer asks questions to elicit information about the area being investigated
		Expert interviews			Conversation with an expert where the interviewer asks questions to elicit information about the area being investigated
		Meta-ethnography			The process of synthesizing qualitative information across studies in a certain area
	Alternative methods	Identifying a predefined policy question	Ryan (1999)		If a certain policy question is being addressed, its characteristics may be predefined so that attributes could be derived from there.
		Theoretical arguments from the literature	Ratcliffe (2000)		Raw data elicited from the literature to reflect key decision criteria which respondents may choose to apply when being asked about allocation decisions.
		Existing health outcome measures	McKenzie et al. (2001)		Utilizing items of existing health outcome measures for developing attributes
		Patient surveys	Moayyedi et al. (2002)		Exploring patient perceptions using a non-DCE survey
		Professional recommendations	Hundley et al. (2001)		Using published guidelines or other similar sources for attribute development
		Expert review	Hall et al. (2002)		Tailored discussion with experts in a certain area, for instance, to obtain comments on findings from a literature review
		Definition of attributes from RCTs	Weston and Fitzgerald (2004)		Noting all significant differences between the components of the arms of a clinical trial
		Brainstorming with experts	Günther et al. (2010)		The group gathers a list of ideas in a creative environment
Stage 2: DATA REDUCTION	Qualitative methods	Thematic analysis	Fitzpatrick et al. (2007)		Identifies themes emerging from the literature and key concepts across interview scripts; data collection discontinues when incoming data do not appear to generate new insights
		Framework	Grewal et al. (2006)		Systematic, comprehensive and rigorous thematic approach to summarize and classify data in a matrix format where columns represent key topics and rows represent individual informants
		Iterative approach	Coast and Horrocks (2007)		Data collection and analysis proceeds concurrently and iterations may not be pre-selected in either size or scope; analysis continues until emerging themes changed the focus of the questioning
		Repertory grid technique	Günther et al. (2010)		Factor analytic approach based on the assumption that the meaning we attach to events or objects defines our subjective reality, and thereby the way we interact with our environment
		Laddering technique	Günther et al. (2010)		Refers to an in-depth, one-on-one interviewing technique used to develop an understanding of how consumers translate the attributes of products into meaningful associations with respect to self, and where the interviewer constantly looks for the subconscious motives behind responses
	Alternative methods	Likert-scale	Essers et al. (2010)		Transforming items identified during data collection into a scale specifying the level of agreement, whereas distance between items is assumed to be equal
		Simple rank ordering	Morgan et al. (2000)		Informants rank attributes in descending order of importance
		Nominal group technique	Sampietro-Colom et al. (2008)		Items identified during data collection have to be scored on a numerical scale from least important to most important
		Frequency	Ratcliffe and Buxton (1999)		Captures how often informants mentioned different attributes during data collection
		Hierarchical information integration	Van Helvoort Postular (2009)		Categorizing the data collected in the first step into several non-overlapping subsets based on logic, empirical evidence, or theory
Stage 3: REMOVING INAPPROPRIATE ATTRIBUTES	Basic criteria	Salient	Ryan and Hughes (1997)	Witt et al. (2009)	Important to patients and/or policymakers
		Plausible		Swancutt et al. (2008)	Feasible to implement and realistically possible to change
		Capable of being traded		Scott (2002)	Respondents are willing to accept more of a specific good or characteristic in compensation for less of another
	Additional criteria	Complete	Coast et al. (2012)		Attributes selected ‘include all those that might be important for an individual in coming to a decision
		Far from latent construct			Attributes are not too close to the latent construct that the DCE is investigating
		Non-dominant			Attributes may be dominant if choices of a group or sub-group of respondents ‘become deterministic rather than stochastic’ because single attributes have too large impact on decisions.
		Manipulable			Attributes should not be intrinsic to a person’s personality and should be experimentally manipulable by intervention
Stage 4: WORDING	Qualitative methods	Pre-testing /piloting	Halme et al. (2009)		Applying qualitative techniques as part of pre-testing and piloting the experiment
		Cognitive interviews	Burge et al. (2004)		Conversation with informants to determine their comprehension of the questions and question format and issues surrounding the selection of attributes
		Think-aloud technique	Cheraghi-Sohi (2007)		Participants thinking aloud while filling out a test version of the DCE questionnaire
	Alternative methods	Researchers’ judgment	Nieboer et al. (2010)		Researcher makes a judgment about the appropriate wording of attributes based on the available data

The process begins with collecting raw data about the area under investigation, for instance, the most important health, non-health, and process outcomes of an intervention. The reviewed literature suggested qualitative methods (e.g., focus groups, patient, and expert interviews) and alternative approaches that may or may not be based on previously defined or published material (e.g., predefined policy questions, theoretical arguments, professional recommendation, etc.). If qualitative methods are used, the researcher obtains a set of unstructured data, which includes all information necessary to define attributes. If alternative methods are used, a long list of potential attributes may be the result of step 1.

After collecting raw data, the next step is to reduce this data into a limited number of attributes. The literature is unclear about what counts as a manageable number, but most studies, e.g., Ryan and Gerard (2003), suggest an upper limit of six or seven attributes to minimize the burden on respondents. Methods reported or suggested for the reduction of data may also referred to as qualitative methods (e.g., thematic analysis, framework, iterative approach, etc.) and alternative techniques (e.g., simple rank ordering, frequency, nominal group technique, etc.).

The third step tests attributes against essential characteristics suggested by the reviewed literature and drops those regarded as “inappropriate.” Ryan (1996) described the basic characteristics for “appropriate” attributes in health-related DCEs, suggesting they should be salient, plausible, and capable of being traded. Coast et al. (2012) added additional criteria which “*ensure that the conceptual framework for random utility theory (…), the psychological basis for DCEs, is not violated”* (p. 734). Accordingly, attributes should (1) include all those which are relevant for an individual’s decision, (2) not be too close to the latent construct that the DCE is investigating, (3) not have too large dominance on a decision so that they become “*deterministic rather than stochastic”* (p. 734), and (4) not be intrinsic to a person’s personality and experimentally manipulable by the intervention. It is possible that attributes can only be tested against some of these characteristics once the results of pre-testing and piloting are available, which also depends on an (initial) wording of attributes. Hence, the third and the fourth step of this process can overlap. Nevertheless, researchers should have a final list of attributes by the end of this step which are confirmed as concepts, but their language may be further refined.

The final step in the process of attribute development aims to ensure that the desired meaning is evoked and that the terminology is understandable for respondents. The literature suggests qualitative techniques (part of pre-testing and piloting, cognitive interviews, think-aloud technique), or researchers’ judgment based on data already available. In some instances (Coast & Horrocks, 2007; Bell et al., 2010), a qualitative method and researchers’ judgment are combined, and some attributes are worded by respondents, whilst others are finalized by the researcher.

## Case study

We applied the proposed four-stage process of attribute development to a planned DCE for eliciting preferences regarding alcohol misuse interventions. As our systematic literature review revealed a multitude of methods suggested for attribute development (Table 1), but without providing much guidance on the actual choice of such methods, testing each of them within our case study was not feasible. Nevertheless, the few recommendations available in the literature imply a tendency toward qualitative approaches. For instance, Coast et al. (2012) state that “*attributes developed through qualitative research are often ‘richer’ than those generated through alternative methods, as they are based on more complex and nuanced data”* (p. 735). We therefore designed our case study so to contrast a “qualitative approach” with an alternative, “mixed approach,” for attribute development. Whilst the first approach employs qualitative techniques throughout the first three stages, the second, alternative, approach employs a mixture of both qualitative and non-qualitative methods. Wording was based on the same method to ensure that, if the same attributes appear, they are also worded identically.

### Methods

For the qualitative approach, expert interviews and focus groups provided raw data about the preferences of individuals affected (positively or negatively) by alcohol misuse interventions. After obtaining ethics approval, we selected 12 experts, including academic researchers, representatives of charities with different missions, and key opinion leaders from the drinking and pub industry. We also conducted five focus groups to account for the preferences of those targeted by alcohol misuse interventions. Emphasis was placed on ensuring diversity in terms of both sociocultural background and religion, as such characteristics were anticipated to play a key role for attitudes toward alcohol consumption. Four focus groups included individuals who may or may not consume alcohol on a regular or irregular basis, whilst one group consisted of people who exclude alcohol consumption from their lives due to religious or personal reasons. Discussions consisted of open questions to assemble the most important impacts of alcohol misuse interventions beyond health. The interviews were transcribed and then reduced to a potential list of attributes using the framework technique and applying the two-stage process described in Grewal et al. (2006). Factors that informants raised as impacts of alcohol misuse interventions were first summarized using informants’ language, followed by further interpretation, grouping, and removal of inappropriate attributes based on the information obtained from the qualitative data.

**Figure 1.**
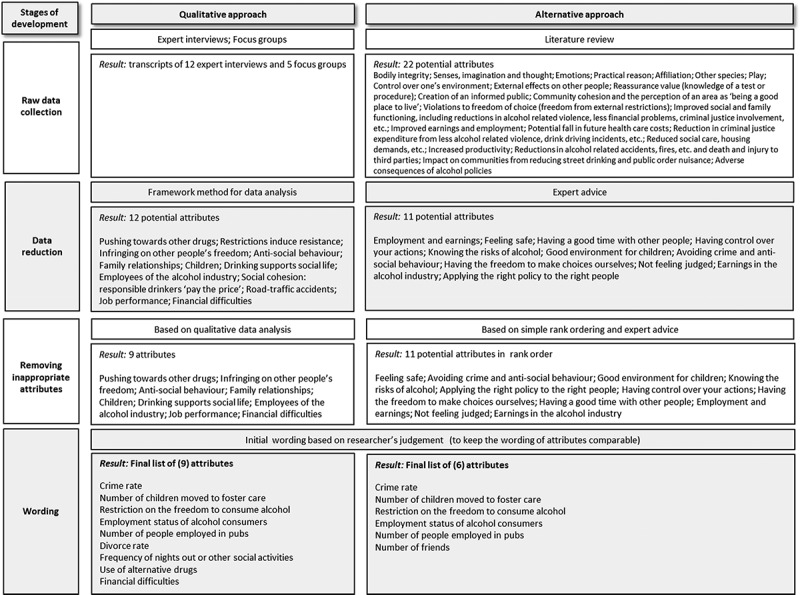
Case study methods and results.

The mixed approach collected data from reviewing the literature related to alcohol misuse in particular and more generally the economic impacts of public health interventions, including the capabilities approach (Nussbaum, 2000). The resulting long list of potential attributes was presented to the experts, but only after concluding open discussions within the qualitative interviews. Experts were asked for their feedback on the concepts identified in the literature, specifically commenting on the importance of each concept to policymakers and general public and their opinion about grouping the concepts found in the literature. Experts’ views were then summarized and applied to the data, resulting in a reduced list of potential attributes. Further, we asked participants after the end of each focus group (described above) to rank order potential attributes according to their importance. During this ranking exercise, participants were also asked for feedback regarding possible correlations and dominant concepts to inform the removal of inappropriate attributes. The methods applied and results obtained within each stage of the attribute development process are displayed in Figure 1.

### Results

The qualitative approach resulted in a large amount of raw data from expert interviews and focus groups in the first step of the proposed framework, whilst we instantly obtained a long list of 22 potential attributes from reviewing the literature within the mixed approach. Consequently, the qualitative approach appeared to be more time and resource consuming than the mixed approach, especially within the first two stages of our proposed framework. However, after the second stage, we obtained a list of 12 potential attributes using qualitative methods and 11 potential attributes from the mixed approach.

These attributes where then tested against essential attribute characteristics (Table 1). The raw data collection method within the qualitative approach ensured that attributes were salient, whilst for the mixed approach, this was achieved through interpreting results from the rank ordering exercise. Attributes were further tested for their plausibility, their capability of being traded, their completeness, their proximity to a latent construct, as well as their dominance and capacity to be manipulated by the intervention. For the qualitative approach, one item (“restrictions induce resistance”) was dropped based on researchers’ judgment for being too intrinsic to a person’s personality and not manipulable by interventions. Further, two items (“social cohesion: responsible drinkers pay the price”; “road-traffic accidents”) were removed as they were often mentioned together with other potential attributes and therefore regarded as closely correlated and not capable of being traded. Within the mixed approach, one attribute (“knowing the risks of alcohol”) was dropped as respondents from the ranking exercise suggested this to be an “umbrella concept” with too large impact on decisions. Two potential attributes (“avoiding crime and anti-social behaviour”; “having control over your actions”) were removed as informants suggested close correlation with other attributes.

The final list comprised nine attributes for the qualitative approach and six attributes for the mixed approach, with a large overlap between both approaches. Five out of six attributes elicited through the mixed approach also appeared through the use of qualitative techniques. The impact of alcohol misuse interventions on the number of friends did only appear in the mixed approach, whilst the impact on the use of alternative drugs, the divorce rate, and financial difficulties were only mentioned in the qualitative approach.

## Discussion

As DCEs are increasingly popular to value outcomes for health economic studies and gradually gain acceptance as an input into policy decisions, results may misguide decision-makers if they are based on an inappropriate set of attributes. Our aim was to inform the progress toward a more systematic approach to attribute development within health-related DCEs. A systematic literature review identified why and how researchers developed or suggested to develop attributes, which methods they used, and how such decisions were justified. Our review confirmed that a systematic approach to attribute development is lacking, identified a range of applicable methods, and proposed a four-stage process for the development of attributes in health-related DCEs. We then conducted a case study in the context of a planned DCE for eliciting preferences regarding alcohol misuse interventions. The dual aim was to test the proposed four-stage process of attribute development and to assess how variation in methods impacts on the results of these experiments.

A number of previous publications aimed at improving the rigor of conducting and reporting health-related DCEs in general (Johnson et al., 2013; Bridges et al., 2011; Lancsar & Louviere, 2008), and the development of attributes for DCEs in particular (Coast et al., 2012; Coast & Horrocks, 2007; Grewal et al., 2006). Coast and Horrocks (2007) state that the rigor with which attributes are developed and reported in health-related DCEs is questionable, an opinion reiterated by Coast et al. (2012). However, the a priori conception in both papers is that qualitative methods are preferable for attribute development, which constrains the applicability of research recommendations to studies using a particular—qualitative—approach. However, more fundamental guidance may be required to move toward a more systematic approach of attribute development for two reasons: first, our systematic review confirms that existing literature provides not just a multitude of methods, both qualitative and non-qualitative, but there is also confusion around the overall process of attribute development, i.e., the individual steps involved to move from raw data to a final set of attributes. Second, though we acknowledge the general tendency toward qualitative methods for developing attributes in DCEs, there are also valid reasons for researchers to deviate from this standard. We therefore contrasted different methods of attribute development within our case study and experienced two potential trade-offs. The first trade-off has already been described by Coast and Horrocks (2007) and relates to the “*purpose of qualitative work (to obtain deep understanding of phenomena) and the essentially reductive aim of describing all the key concepts of care in as few attributes as possible*” (p. 29). The second, more general trade-off exists between the application of qualitative methods within the process of attribute development and their implications on the analytic resources required to conduct a DCE.

It is not possible to answer the question whether different combinations of methods within our proposed four-stage process would have resulted in different sets of attributes. Further, correlation between the results of both approaches may relate to the fact that the same researchers carried out both the qualitative and the mixed approach and that focus group members and experts consulted within the qualitative approach were the same individuals who provided specialist advice and ranked attributes in the mixed-methods approach. Nevertheless, our proposed four-stage process to attribute development was particularly useful for the conduct of our case study, as it provided a clear structure to a key aspect in the design of DCEs, which thus far, unambiguously lacks a systematic approach. Finally, though inextricably tied to the development of attributes, we did not address level selection for attributes in this study. We believe that level selection is an issue which should be addressed alongside the four stages of attribute development which we propose here. However, further research is required.

## Conclusion

With this article, we hope to provide a reference point for the design and conduct of future health-related DCEs We propose a four-stage process to inform the progress toward a more systematic approach to attribute development. Future research should look into the implications of mixing different methods for the development of attributes within DCEs, and how to incorporate level selection into this process.
